# Percentage Amplitude of Fluctuation Alterations in Multiple Frequency Bands in Patients With Transient Ischemic Attack: A Resting-State fMRI Study

**DOI:** 10.1155/np/8110535

**Published:** 2025-05-31

**Authors:** Xinyun Li, Wei Zou, Fengjia Ni, Kelin He, Yingying Gao, Zhiyong Zhao, Yulin Song, Ruijie Ma

**Affiliations:** ^1^Hangzhou Medical College Rehabilitation School, Hangzhou, Zhejiang, China; ^2^Zhejiang Chinese Medical University Affiliated Third Hospital, Department of Acupuncture, Hangzhou, Zhejiang, China; ^3^Department of Radiology, Foresea Life Insurance Nanning Hospital, Nanning, China; ^4^The First Hospital of Xinjiang Production and Construction Group, Aksu, Xinjiang, China; ^5^Children's Hospital, Zhejiang University School of Medicine, National Clinical Research Center for Child Health, Zhejiang University, Hangzhou, Zhejiang, China; ^6^Department of Neurology, Anshan Changda Hospital, Anshan, China

**Keywords:** frequency-specific, percent amplitude of fluctuation (PerAF), resting-state functional magnetic resonance imaging, transient ischemic attack

## Abstract

**Purpose:** This study aims to investigate functional abnormalities in transient ischemic attack (TIA) patients compared to healthy controls (HCs) using percent amplitude of fluctuation (PerAF) across multiple frequency bands derived from resting-state functional magnetic resonance imaging (rs-fMRI).

**Methods:** We scanned 48 TIA patients and 41 HCs using rs-fMRI and high-resolution T1-weighted brain images. Both PerAF and modified PerAF (mPerAF) were utilized for comparative analysis across the typical frequency band (0.01–0.08 Hz) and two subfrequency bands: slow-4 (0.027–0.073 Hz) and slow-5 (0.01–0.027 Hz). Two-sample *t*-tests were conducted to assess group differences, with multiple comparisons correction using Gaussian random field (GRF) methods.

**Results:** Compared to HCs, TIA patients exhibited significantly lower PerAF in the right inferior frontal triangular gyrus in both the typical and slow-5 bands. Additionally, reductions were observed in the right superior frontal medial gyrus in the slow-4 band and the left middle temporal gyrus in the slow-5 band. No significant differences were observed in mPerAF.

**Conclusion:** These findings suggest a significant impact of TIA on multiple brain regions, with frequency-specific alterations in PerAF, providing novel insights into the underlying mechanisms of TIA.

## 1. Introduction

Transient ischemic attack (TIA) is a serious, reversible neurological condition caused by focal cerebral hypoperfusion [1, 2]. It is acknowledged that TIA is a significant risk factor for subsequent stroke or silent stroke [3]. Given up to 80% of strokes after TIA are preventable [4], investigating brain abnormalities associated with TIA is crucial, as it offers the best opportunity for early stroke intervention.

Resting-state functional magnetic resonance imaging (rs-fMRI) measures blood oxygenation level-dependent (BOLD) signals, which reflect spontaneous neuronal activity through low-frequency fluctuations [5–8]. Rs-fMRI has become a vital tool for probing neurophysiological mechanisms in TIA [9–12]. For example, two studies observed altered functional connectivity (FC) and Granger causality analysis across various brain networks in TIA patients [11, 12]. Additionally, Lv et al. used rs-fMRI to reveal the functional abnormalities in TIA patients based on amplitude of low-frequency fluctuations (ALFFs), fractional ALFF (fALFF), and degree centrality [9], and Ma et al. [10] found dynamic alterations in resting-state local metrics, such as ALFF, fALFF, and regional homogeneity, which distinguished TIA patients from healthy controls (HCs). Although ALFF and fALFF show superior performance in exploring neural activity in TIA, ALFF is a scale-dependent parameter, and fALFF may be affected by confounding mixture from voxel-specific fluctuation amplitude [9, 10, 13].

Percent amplitude of fluctuation (PerAF) quantifies the proportion of BOLD fluctuation relative to the mean BOLD signal intensity at each time point, averaged over the entire time series. It provides a scale-independent metric similar to percent signal change in task-based fMRI [13, 14]. As a recent voxel-level metric, PerAF has shown superior reliability and sensitivity compared to ALFF and fALFF in test–retest reliability analysis [13, 15]. The PerAF approach has been applied to investigate neural mechanisms in various diseases [16], suggesting its potential for exploring functional changes in the brains of TIA patients.

Most rs-fMRI studies have focused on low-frequency oscillations within the typical frequency band of 0.01–0.08 Hz, believed to reflect spontaneous neuronal activities [8, 12, 17]. Recent research has shown that intrinsic neuronal activity patterns are sensitive to specific frequency bands, with low-frequency oscillation amplitudes in different bands reflecting meaningful differences across brain regions [18]. Decomposing rs-fMRI low-frequency oscillations into five distinct bands, slow-6 (0–0.01 Hz), slow-5 (0.01–0.027 Hz), slow-4 (0.027–0.073 Hz), slow-3 (0.073–0.198 Hz), and slow-2 (0.198–0.25 Hz), reveals that oscillation amplitudes in the slow-4 and slow-5 bands are predominantly observed in gray matter [19]. In contrast, signals in the slow-6, slow-3, and slow-2 bands reflect low-frequency drift, white matter signals, and high-frequency physiological noise, respectively [18–20]. Frequency-dependent changes in low-frequency oscillations have been reported in various diseases, including mild cognitive impairment [21], Parkinson's disease [22, 23], and schizophrenia [24]. Zhu et al. [25] recently demonstrated that parietal lobe abnormalities in stroke patients were frequency-dependent compared to HCs, suggesting that rs-fMRI studies of stroke should consider frequency effects when measuring intrinsic brain activity. However, no studies have yet explored whether altered low-frequency oscillations in TIA patients are associated with specific frequency bands.

In this study, we employed PerAF to explore brain region abnormalities in TIA patients compared to HCs, extending this approach to examine spontaneous neuronal activities in different low-frequency oscillation bands (slow-5 [0.01–0.027 Hz], slow-4 [0.027–0.073 Hz], and the typical band [0.01–0.08 Hz]). We hypothesized that TIA patients would exhibit altered PerAF in the typical band compared to HCs, and we expected to identify new regions within the subfrequency bands that differed from those in the typical band.

## 2. Materials and Methods

### 2.1. Subjects

A total of 51 TIA patients were initially recruited from the Department of Neurology at Anshan Changda Hospital from April 2015 to June 2016, based on clinical evaluations by neurologist. Additionally, 41 HCs without physical illnesses or history of psychiatric or neurological disorders were recruited from the local community. The inclusion criteria for the patients were (1) age between 45 and 80 years; (2) MRI scan time within 72 h after the onset of TIA; and (3) a clinical diagnosis of TIA, defined as acute, transient, and localized neurological dysfunction lasting no longer than 24 h. Exclusion criteria for all participants included (1) any neuropsychiatric comorbidity (e.g., depression, epilepsy, brain tumors, brain trauma, and drug or alcohol abuse); (2) clinically significant or unstable medical conditions; (3) contraindication for MRI; (4) pregnant or lactation; and (5) aphasia either prior to or following the stroke.

The study was approved by the Ethics Committee of the Center for Cognition and Brain Disorders at Hangzhou Normal University (Ethical Number: 20160412), and all participants provided written informed consent.

### 2.2. MRI Data Acquisition

MRI data were acquired on a GE MR-750 3.0T scanner (GE Medical Systems, Inc., Waukesha, WI, USA) at Anshan Changda Hospital. The time interval between the last TIA attack and subsequent MRI scanning ranged from 0.25 to 6 days for the patients. During data acquisition, participants were instructed to remain awake, relax with their eyes closed, and minimize motion.

Resting-state fMRI (rs-fMRI) data were obtained using an echo-planar imaging sequence with the following parameters: 43 axial slices, TE = 30 ms, TR = 2000 ms, FA = 60°, slice thickness/gap = 3.2/0 mm, and matrix size = 64 × 64. The in-plane resolution was 3.44 mm × 3.44 mm, comprising 240 contiguous EPI functional volumes, with a total session duration of ~8 min.

T1-weighted images were captured initially, excluding any intracranial organic diseases, such as tumor-like lesions. T1 images were acquired using a T1-weighted sagittal 3D magnetization-prepared rapid gradient echo (MPRAGE) sequence: 176 sagittal slices, TR = 8100 ms, TE = 3.1 ms, matrix = 256 × 256, voxel size = 1 mm × 1 mm × 1 mm, and thickness/gap = 1/0 mm. This session lasted ~5 min.

Two patients and one patient were excluded from further analysis due to incomplete brain coverage in the rs-fMRI scan and loss of the 3D T1 image, respectively. As a result, the final analysis included 48 TIA patients and 41 age- and sex-matched HCs.

### 2.3. Data Preprocessing

rs-fMRI data were processed using Data Processing & Analysis for Brain Imaging (DPABI V8.2) toolbox (https://rfmri.org/DPABI) [26], based on Statistical Parametric Mapping (SPM12, http://www.fil.ion.ucl.ac.uk/spm), running on Matlab2017b (MathWorks, Natick, MA, USA). The preprocessing steps were as follows: (1) removal of the first 10 time points to allow longitudinal magnetization to reach a steady state; (2) slice-timing correction to adjust for acquisition time differences between slices; (3) head motion correction; (4) spatial normalization to the Montreal Neurological Institute (MNI) space using DARTEL segmentation of structural images (resampling voxel size = 3 × 3 × 3 mm^3^); (5) spatial smoothing with an isotropic Gaussian kernel with a full width at half maximum (FWHM) of 6 mm; (6) removal of linear trends from the time course; (7) regression of head motion effects using Friston's 24 parameters, mean white matter, global mean signal, and cerebrospinal fluid signals from the fMRI data [27]; and (8) bandpass filtering 0.01–0.08 (typical), 0.01–0.027 Hz (slow-5), and 0.027–0.073 Hz (slow-4), respectively. No patients were excluded due to excessive head motion (defined as more than 3.0 mm of maximum translation in any direction or 3.0 of maximum rotation during the scan).

Following preprocessing, two analytic methods, including PerAF and modified PerAF (mPerAF), were applied to the whole-brain fMRI data at the voxel level.

### 2.4. PerAF Calculation

PerAF for each voxel was calculated in an in-house code based on MATLAB as follows [13, 28, 29]:

(1) $$\begin{array}{c} \text{PerAF}=\frac{1}{n}\sum_{i=1}^{n}\left|\frac{X_{i}-\mu }{\mu }\right|\times 100\%\;, \end{array}$$

(2) $$\begin{array}{c} \mu =\frac{1}{n}\sum_{i=1}^{n}X_{i}, \end{array}$$

where *X*_*i*_ is the signal intensity of the *i*_*th*_ time point, *n* is the total number of time points of the time series, and *μ* is the mean value of the time series. Briefly, we assume there is a resting-state time series with *n* time points (*n* = 230 in our study). First, the signal intensity values of each voxel were averaged across all time points as defined *μ* (Equation (2)). Then, for each time point (*X*_*i*_), the value of *μ* was subtracted, and the result was divided by *μ*. The estimated values were summed, and the summed results were averaged over the number of time points (Equation (1)). Meanwhile, the PerAF of each voxel was also divided by the global mean PerAF of each participant, yielding both PerAF and mPerAF.

### 2.5. Statistical Analysis

Demographic differences between TIA patients and HCs were analyzed using Statistical Package for the Social Sciences (SPSS22.0, IBM, Armonk, NY, USA). Age differences were assessed using two-sample *t*-tests, and sex differences were analyzed using the Pearson chi-square test.

Two-sample *t*-tests were used to compare PerAF values between TIA patients and HCs. The multiple comparisons of resulting T-maps were employed Gaussian random field (GRF) theory at voxel-wise *p* < 0.001 and cluster-wise *p* < 0.05.

## 3. Results

### 3.1. Demographics Characteristics of Participants

As shown in Table 1, there were no significant differences between TIA patients and HCs in sex (*p* = 0.67) and age (*p* = 0.18).

### 3.2. PerAF Results

**Figure 1:**
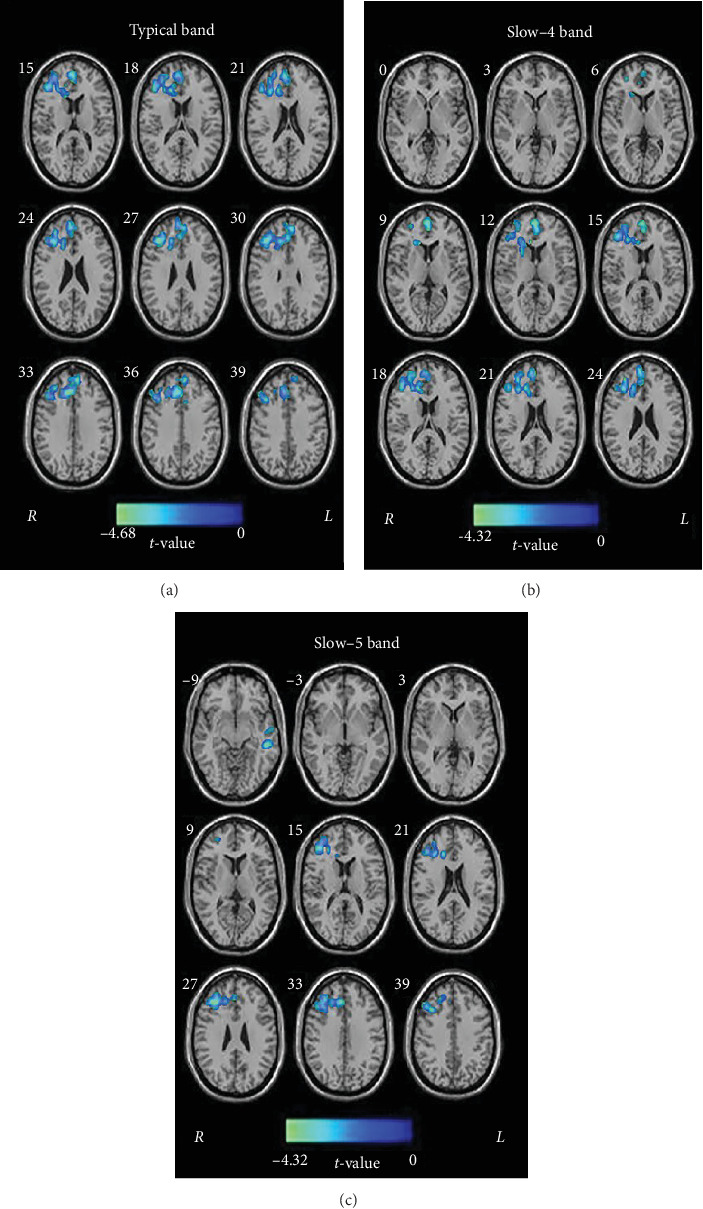
Differences between the two groups in PerAF in the typical band (A), slow-4 band (B), and slow-5 band (C). Brighter color indicates lower PerAF in the TIA group compared with the HC group (*p* < 0.05, corrected). L, left; R, right.

Compared to HCs, TIA patients exhibited frequency-dependent alterations in PerAF. Specifically, in the typical band, TIA patients showed decreased PerAF in the right inferior triangular frontal gyrus (IFGtri). In the slow-4 band, TIA patients had decreased PerAF in the right medial superior frontal gyrus (SFGmed). Furthermore, in the slow-5 band, TIA patients exhibited lower PerAF in left middle temporal gyrus (MTG) and the right IFGtri compared to HCs (Table 2, Figure 1).

However, there was no significant differences between TIA and HCs in mPerAF.

## 4. Discussion

To our knowledge, this study is the first to investigate frequency-dependent effects on PerAF in patients with TIA. Our findings demonstrate that TIA patients exhibit reduced PerAF in specific brain regions across different frequency bands. In the typical band, decreased PerAF was observed in the right IFGtri, while the slow-4 band showed reduced PerAF in the right SFGmed. Additionally, in the slow-5 band, TIA patients exhibited lower PerAF in the left MTG and right IFGtri. These results support our hypothesis that spontaneous brain activity after TIA is influenced by specific frequency bands.

Compared to HCs, TIA patients demonstrated decreased PerAF in the right IFGtri in both the typical and slow-5 bands. The IFGtri is involved in diverse cognitive functions, including object recognition [30, 31], attention, motor inhibition, and language processing [17, 32, 33]. Meanwhile, previous studies have reported decreased degree centrality in right inferior frontal gyrus (IFG) of TIA patients [9], as well as reduced FC between the inferior frontal cortex and the dorsal attention network in poststroke memory dysfunction [34]. Moreover, activation of the right IFG may be crucial for language performance in patients with aphasia following left hemispheric strokes [35]. Thus, the reduced PerAF in the right IFGtri might reflect functional abnormalities related to attention and language deficits in TIA patients [28, 29].

Furthermore, TIA patients exhibited decreased PerAF in the left MTG in the slow-5 band compared to HCs. The MTG is essential for cognitive functions such as language and semantic memory processing, as well as visual perception [12, 36–38]. Previous research has shown decreased FC in the left MTG within the default mode network (DMN) in TIA patients [12, 39]. Despite the transient nature of clinical symptoms, TIA is associated with impairments in various cognitive domains, including executive function and information processing speed [39, 40]. Thus, the reduced PerAF in the left MTG may be linked to language processing difficulties observed in TIA patients.

In the slow-4 band, decreased PerAF was found in the right SFGmed in TIA patients. The SFGmed is a key component of the cortical-basal ganglia loop, which is involved in voluntary movement and complex motor activities, including language initiation [41, 42]. This region integrates afferent inputs and efferent outputs from the motor cortex, facilitating complex movement sequences and memory organization [42]. Additionally, the right SFGmed is connected to the primary motor cortex, premotor area, and anterior cingulate cortex [43–45]. Therefore, the decreased PerAF observed in TIA patients in this region may be associated with impaired motor function and motor control [43–45]. For example, TIA patients often experience involuntary muscle spasms. The right SFGmed is also considered a key node of the DMN [46], which maintains self-referential activity and environmental monitoring [47, 48]. The reduced PerAF in the right SFGmed in TIA patients may disrupt these functions, affecting posture control and leading to involuntary movements. Thus, we speculate cautiously that the DMN may play a role in TIA, warranting further investigation as a potential biomarker.

Previous studies have reported frequency-dependent alterations in brain activity across various diseases [19, 49–51]. For example, patients with Alzheimer's disease showed widespread abnormalities in intrinsic brain activity, with greater alterations in the slow-5 band compared to the slow-4 band [49]. Similarly, Zhao et al. [19] found more functional abnormalities in motor-related regions in both the slow-5 and slow-4 bands compared to typical bands. Hoptman et al. [50] demonstrated that abnormalities in low-frequency oscillation amplitudes were particularly prominent in the slow-4 band in schizophrenia. Additionally, studies on children with attention-deficit hyperactivity disorder suggested that the slow-4 band provided more diagnostic information than other frequency bands [51]. In our study, we observed that the right SFGmed showed greater sensitivity to the slow-4 band, while the left MTG exhibited more prominent changes in the slow-5 band in TIA patients. These findings suggest that oscillations in different frequency bands may reflect varying levels of alteration in specific brain regions. Therefore, frequency-dependent analysis could detect distinct intrinsic brain activities, offering a valuable method for more comprehensively exploring brain activity in TIA.

Despite these promising findings, the study has some limitations. First, the data are descriptive, requiring further clinical validation to establish the reliability of PerAF as a biomarker for TIA. For example, future studies should correlate PerAF with clinical assessments. Second, the absence of cognitive data for TIA patients limits our ability to explore the relationship between functional brain alterations and cognitive dysfunction associated with TIA. Finally, the lack of longitudinal MRI data prevents us from examining how PerAF changes over time as TIA progresses.

In conclusion, the PerAF method offers a valuable approach for detecting subtle but significant changes in brain function in TIA patients. The frequency-specific alterations observed across different brain regions provide new insights into the neural mechanisms affected by TIA, potentially enhancing our understanding and management of this condition.

## Figures and Tables

**Table 1:** Demographics of all participants.

	TIA (*n* = 48)	HCs (*n* = 41)	*p*-Value
Age (years)	57.604 ± 9.778	55.024 ± 8.033	0.182^a^
Sex (M/F)	37/11	30/11	0.670^b^

^a^Data were obtained using two-sample two-side *t*-tests.

^b^Data were obtained using Pearson chi-square tests.

**Table 2:** Regions showing significantly different PerAF in the TIA group compared with the HC group.

Bands (Hz)	Brain regions (AAL)	Cluster size	MNI coordinates			Peak *t*-value
			*X*	*Y*	*Z*	
0.01–0.08	IFGtri _R	993	39	33	27	−4.681

0.027–0.73	SFGmed_R	898	3	57	12	−4.319

0.01–0.027	MTG_L	300	−51	−30	–9	−4.066
	IFGtri_R	469	39	33	27	−4.655

Abbreviations: AAL, anatomical automatic labeling; IFGtri, inferior triangular frontal gyrus; L, left; MTG, middle temporal gyrus; R, right; SFGmed, medial superior frontal gyrus.

## Data Availability

The datasets generated and analyzed during the current study are available from the corresponding author upon reasonable request, subject to ethical approvals and data sharing agreements. All data were anonymized prior to analysis to protect the participant privacy and relevant institutional guidelines. (1) Data storage and accessibility: Raw and processed neuroimaging data (rs-fMRI and T1-weighted images) are stored in institutional secure servers at Anshan Changda Hospital and Hangzhou Normal University. Access is restricted to authorized researchers via role-based authentication and requires approval from the Ethics Committee of the Center for Cognition and Brain Disorders, Hangzhou Normal University (Ethical Number: 20160412). (2) Data retention and deletion: Data will be retained for 5 years postpublication to facilitate verification and secondary analyses. After this period, de-identified datasets will be securely destroyed following institutional protocols. (3) Data sharing policy: Researchers requesting access must submit a formal proposal outlining their intended use, methodology, and ethical approvals. Collaborative agreements may be required for multisite analyses.
